# Scaling severe acute malnutrition treatment with community health workers: a geospatial coverage analysis in rural Mali

**DOI:** 10.1186/s12960-022-00771-8

**Published:** 2022-10-21

**Authors:** Pilar Charle-Cuéllar, Lidia Espí-Verdú, Juan Goyanes, Magloire Bunkembo, Salimata Samake, Mamadou Traore, Adama Balla Coulibaly, Aly Landouré, Fatou Diawara, Abdias Ogobara Dougnon, Antonio Vargas, Noemí López-Ejeda

**Affiliations:** 1 Action Against Hunger Spain, C/ Duque de Sevilla No. 3. 28002, Madrid, Spain; 2 Action Against Hunger Mali, BP 2562, Bamako, Mali; 3 Nutrition Direction of the Ministry of Hygiene and Public Health, BP 232, Bamako, Mali; 4 Institut National de Recherche en Santé Publique, BP 235, Bamako, Mali; 5 Action Against Hunger West Africa Regional Office, BP 29621, Dakar, Senegal; 6 EPINUT Research Group (Ref. 920325), Faculty of Biological Sciences, Department of Biodiversity, Ecology and Evolution, Unit of Physical Anthropology, Complutense University of Madrid, Jose Antonio Novais, 12, 8th Floor, 28040 Madrid, Spain

**Keywords:** Severe acute malnutrition (SAM), Community health workers (CHW), Integrated Community Case Management (iCCM), Coverage, Geographical accessibility, Simplified approaches

## Abstract

**Background:**

In 2015, the Ministry of Health in Mali included the treatment of severe acute malnutrition (SAM) into the package of activities of the integrated Community Case Management (iCCM). This paper aims to analyze the impact of including community health workers (CHWs) as treatment providers outside the Health Facilities (HFs) on the coverage of SAM treatment when scaling up the intervention in the three largest districts of the Kayes Region in Mali.

**Methods:**

A baseline coverage assessment was conducted in August 2017 in the three districts before the CHWs started treating SAM. The end-line assessment was conducted one year later, in August 2018. Coverage was assessed by the standardized methodology called Semi-Quantitative Evaluation of Access and Coverage (SQUEAC). The primary outcome was treatment coverage and other variables evaluated were the geographical distribution of the HFs, CHW’s sites and overlapping between both health providers, the estimation of children with geographical access to health care and the estimation of children screened for acute malnutrition in their communities.

**Results:**

Treatment coverage increased in Kayes (28.7–57.1%) and Bafoulabé (20.4–61.1%) but did not in Kita (28.4–28.5%). The decentralization of treatment has not had the same impact on coverage in all districts, with significant differences. The geospatial analyses showed that Kita had a high proportion of overlap between HFs and/or CHWs 48.7% (39.2–58.2), a high proportion of children without geographical access to health care 70.4% (70.1–70.6), and a high proportion of children not screened for SAM in their communities 52.2% (51.9–52.5).

**Conclusions:**

Working with CHWs in SAM increases treatment coverage, but other critical aspects need to be considered by policymakers if this intervention model is intended to be scaled up at the country level. To improve families’ access to nutritional health care, before establishing decentralized treatment in a whole region it must be considered the geographical location of CHWs. This previous assessment will avoid overlap among health providers and ensure the coverage of all unserved areas according to their population densities need.

**Trial registration:** ISRCTN registry with ID 1990746. https://doi.org/10.1186/ISRCTN14990746

## Introduction

In Africa, 13.6 million children suffer from severe acute malnutrition (SAM), a form of undernutrition with an increased risk of death [1, 2]. Mali is one of the Sahel countries that repeatedly faces a nutritional crisis every year. This situation is exacerbated by various factors related to household food insecurity and access to quality health care [3]. The results of the 2018 Standardized Monitoring and Assessment of Relief and Transitions (SMART) survey showed that the prevalence of global acute malnutrition (GAM) in children under five years of age was 10% (95% CI 9.1–11.0), and the prevalence of SAM was 2.0% (95% CI 1.6–2.4) [4]. The threshold of 10% prevalence of GAM classifies this country as having a high prevalence of the disease according to international standards [5]. This form of malnutrition together with diarrhea is one of the leading causes of death in children under five in Mali [6]. According to the last coverage survey performed in Mali, at a country level, only the 22.3% (95% CI 16.7–27.6) of children with SAM have access to the treatment they need, and this figure rises to 24.5% (95% CI 18.1–30.6) in the Kayes region [7]. This proportion is under the 50% considered as acceptable in the international Sphere standards for the Community Management of Acute Malnutrition (CMAM) programs [8]. The main barriers to treatment revealed in the survey were caregivers’ lack of knowledge about the child’s illness and the distance and lack of money to access health facilities (HFs) [7].

For CMAM programs, effectiveness and treatment coverage are strongly linked. Even if a program achieves good clinical outcomes, high cured proportion and low deaths, the ultimate impact is minimal if it only reaches low levels of coverage. When high effectiveness and high coverage are combined, a CMAM program meets the needs of the target population [9, 10]. Some barriers to SAM treatment are linked with the health service delivery model itself. Even if SAM treatment is available and integrated into the health system, HFs are still located far from communities. This can mean that mothers and caregivers may decide not to attend HFs for SAM treatment because of the direct and indirect costs (payment of transport, loss of income, insecurity) that can jeopardize the entire family’s livelihood [11, 12].

After reviewing the evidence obtained during a pilot study carried out in the District of Kita, the Malian government modified its primary health care policy in 2015 (*Soins Essentiels dans la Communauté Guide*) [13, 14]. This policy included SAM treatment within the package of integrated Community Case Management (iCCM) activities that community health workers (CHWs) were required to carry out in their villages. The World Health Organization (WHO) published the Global Action Plan 2020 to Accelerate Progress in Preventing and Managing Child Wasting, in which they encouraged strengthening the integration of early detection and treatment for wasting with CHWs as part of routine community health care services to increase coverage of the disease [15].

At the international level, there is already evidence about the effectiveness of CHWs in treating SAM in different locations, including Mali [16–18]. However, less evidence is found on which factors can influence coverage of SAM treatment if this decentralized model of treatment with CHWs is taken to scale. [17, 19]. Our study aims to analyze the impact of including CHWs as treatment providers outside the HFs on the coverage of SAM treatment when scaling up the intervention in the three largest districts of the Kayes Region in Mali, examining the possible influence of other related factors.

## Materials and methods

### Location and study design

The intervention was conducted between October 2017 and October 2018 to compare the coverage and effectiveness obtained with CHWs treating children 6–59 months for uncomplicated SAM under different levels of supportive supervision in the three largest districts of the Kayes region. The CHWs of Kita District received a high level of supervision (standard country’s supervision on primary health care plus nutrition-specific supervision monthly assured by Action Against Hunger staff), Kayes District received a mild level of supervision (standard country’s supervision monthly assured by Action Against Hunger staff without nutrition-specific supervision) and Bafoulabé District considered the control arm with the standard supervision from the health authorities without any extra support. Results on the effectiveness achieved by CHWs under those different supervision models were published elsewhere showing that the level of supervision has a differential effect on the quality of treatment [20]. It is not expected that the different supervision models will have any effect on treatment coverage.

The main objective of the present study was to comparatively evaluate the coverage of SAM treatment in those three districts of the Kayes region, before and after including the CHWs as new SAM treatment providers supporting the HFs. In addition, other variables that could potentially influence coverage were assessed in the three districts: (1) the geographical distribution of the HFs and CHWs sites and the overlap between the areas covered by both health care providers; (2) the estimate of the number of children under five years of age with geographical access to health care; (3) the estimate of children under five screened for SAM by community health volunteers (called *Relais Communautaires*).

The study was approved by the Ethical Committee of the National Institute of Public Health Research (INRSP) in Bamako (Decision no. 13/2017CE-INRSP). The study protocol was registered at the ISRCTN https://doi.org/10.1186/ISRCTN14990746.

### Treatment coverage assessment

A baseline coverage assessment was conducted in August 2017 in the three districts before the CHWs started treating SAM. The end-line assessment was conducted one year later. The coverage of SAM treatment was defined as the proportion of 6–59 months children eligible for treatment service compared to the number of 6–59 children who actually received that service. Coverage was assessed by the standardized methodology Semi-Quantitative Evaluation of Access and Coverage (SQUEAC), which provides an in-depth investigation and analysis of barriers and boosters of access [21]. The assessment was conducted in three stages: Stage 1, to identify areas of low and high coverage as well as reasons for coverage failure using routine program quantitative data and qualitative data collected before the survey; Stage 2, to confirm the location of areas of high and low coverage and the reasons for coverage failure identified in Stage 1 through small surveys; Stage 3, to provide an estimate of overall program coverage using Bayesian techniques.

A single coverage indicator was used to estimate the coverage of SAM treatment, which also includes the recovering cases both within and outside of the program using the following formula:

$${\text{Single coverage }} = \, \left( {{\text{Cin}} + {\text{ Rin}}} \right)/ \, \left( {{\text{Cin}} + {\text{Rin}} + {\text{Cout}} + {\text{Rout}}} \right),$$

where Cin = current SAM cases in the program; Cout = current SAM cases, not in the program; Rin = recovering SAM cases in the program; Rout = recovering SAM cases, not in the program.

To analyze access barriers to SAM treatment, semi-structured interviews and discussion groups were organized with the CHWs and nurses, community leaders and mothers/caregivers.

### Population data

Mali’s administrative structure comprises different levels ranging from the highest level to the lowest level. The highest level is the first-level administrative division, which refers to regions. The intermediate level is the second-level administrative division, called district. And the lowest level is the third-level administrative division composed of communes. A series of steps were taken to obtain the total population by the district at the third-level administrative division. First, the 2018 United Nations Office for the Coordination of Humanitarian Affairs (OCHA) projections were assumed based on the 2009 census to obtain population data by villages [22]. Then, we applied a population growth rate of 3% from the United Nations rapport “World population perspectives”, to obtain the total population in 2019 [23]. Next, the population under five years of age was estimated at 20% of the total, and the proportion of children with SAM at 2% (estimates used in the national SMART survey of 2018) [4]. Accordingly, the under-five population obtained for the three districts was: 109,927 in Kita, 130,423 children in Kayes, and 31,494 in Bafoulabé.

### Ancillary data sources

According to the Ministry of Health's policy in Mali, CHWs must be located at more than 5 km from the referring HF, and they must cover a population of approximately 700 people within a radius of 3 km around the village site [13].

Data of children screened for SAM come from the District's Activity Reports, including routine screening performed by community health volunteers and mass screening campaigns conducted during the study period. A SAM case was defined as a child between 6 and 59 months with a middle-upper arm circumference (MUAC) < 115 mm and/or edema. All the children identified with SAM in the villages were referred to the nearest HF or CHW to receive treatment.

### Mapping and geospatial analysis

ArcGIS Desktop 10.8 was used for mapping and geospatial analysis of factors influencing SAM treatment coverage achieved by adding CHWs as treatment providers [24]. The three health district boundaries in the maps were established using the information provided in the Humanitarian Data Exchange [25]. The 112 HFs and 152 CHWs in the area were georeferenced to analyze the spatial distribution of health services delivery. From that number of health care providers, Kita district has 38 HFs and 75 CHWs, Kayes has 52 HFs and 43 CHWs, and Bafoulabé, 22 HFs, and 34 CHWs. The total area in km^2^ covered by the health care services and the area not covered by either HF or CHWs were also calculated.

The influential variables with a possible impact on treatment coverage were analyzed. First, the overlap between areas covered by the treatment providers was defined as an HF located less than 5 km from other HFs, a CHW located less than 5 km from the HF, or a CHW located less than 3 km from another CHW. Second, the proportion of children under 5 years without geographical access to any health provider was defined as the children living more than 5 km to the nearest HF or CHW place from the estimated total children under five living in the health area. Third, the proportion of children under five years not covered by active screening, defined as children under five who have not been screened for SAM in their communities from the estimated total children under five living in the health area.

### Statistical analysis

The statistical analysis was performed with the online software Epitools (https://epitools.ausvet.com.au/). Proportions were compared by the Chi-squared test applying Yates correction when expected cases were less than 5 in more than 20% cells. The Mantel–Haenszel Chi-square test was applied to compare the final treatment coverage between groups adjusted to the initial coverage providing the associated odds ratio (OR). A 95% confidence level was applied in all analyses, considering significant *p*-values below 0.05.

## Results

The baseline and end-line coverage assessment results can be found in Table 1. The SAM treatment coverage significantly increased in Kayes and Bafoulabé districts during the intervention period, but no difference was found in the District of Kita. Initial coverage did not differ between districts when they were compared by pair. A comparative analysis of final coverage adjusted for baseline coverage found significant differences between Kita and Kayes (OR: 0.41 [0.11–0.48], *p* < 0.001) and Kita vs. Bafoulabé (OR: 0.44 [0.26–0.76], *p* = 0.004). The difference was not significant comparing Kayes vs. Bafoulabé (OR: 1.11 [0.65–1.89], *p* = 0.814).

**Table 1:** SAM treatment coverage at the beginning of the study and after 12 months of the intervention with CHWs treating SAM in the three largest districts of Kayes Region in Mali

	^a^Kita District % (95% CI) High supervision	^b^Kayes District % (95% CI) Light supervision	^c^Bafoulabé District % (95% CI) Not supported supervision	Comparison (*p* value)
Baseline (August 2017)	28.4 (19.9–39.2)	28.7 (20.6–38.6)	20.4 (12.9–30.8)	a–b: 0.953 a–c: 0.396 b–c: 0.344
End-line (August 2018)	28.5 (20.6–37.9)	57.1 (48.1–65.7)	61.1 (51.4–70.1)	a–b: < 0.001 a–c: < 0.001 b–c: 0.694
Difference	+ 0.1%	+ 28.4%	+ 40.7%	
Comparison (*p* value)	0.934	< 0.001	< 0.001	

The letters a, b and c refer to each of the districts to summarize which districts are being compared in the final comparison column

Barriers to SAM treatment, identified in the qualitative part of the baseline coverage survey, were similar in the three districts: caregivers’ lack of knowledge of symptoms, causes and effects of SAM, long distance to the HF, defaulters due to high cost of transport to the HF, lack of SAM screening in the community, instability of CHWs, treatment provided by traditional healers and stock-out of Ready-to-Use Therapeutic Food (RUTF). The end-line assessment shows an improvement in the Kayes district in two of the access barriers: a decrease in the number of defaulters and an increase in screening campaigns at the community level; in Bafoulabe, the number of families citing distance to HF as a problem has also decreased and screening campaigns at the community were done; and in Kita district, the barriers to accessing treatment were the same in the final survey comparing with the baseline one. The barrier of caregivers’ lack of knowledge of malnutrition was still in the three groups. A summary of the SQUEAC survey is available in the supplementary material.

**Fig. 1:**
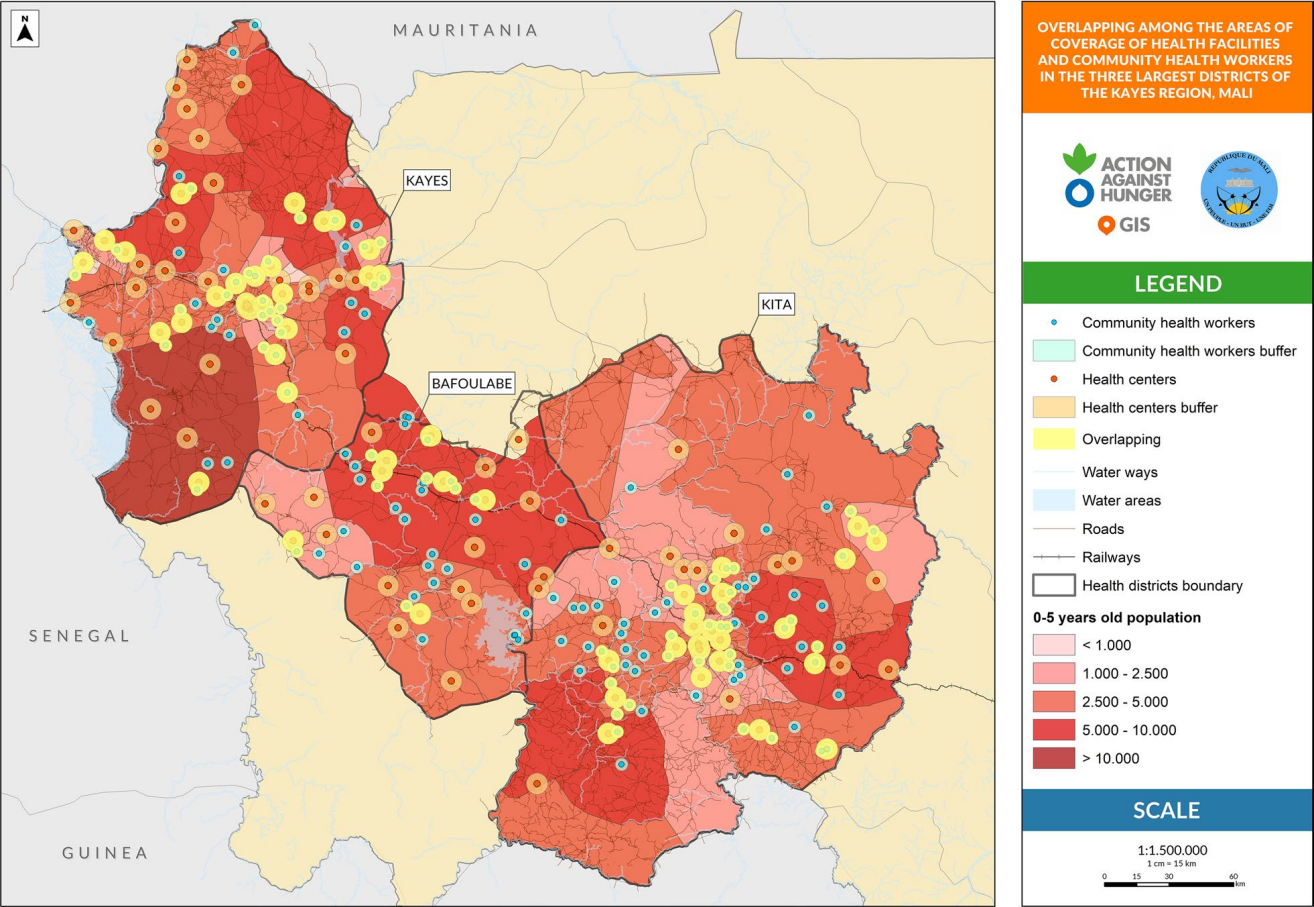
Overlap between areas covered by health facilities and Community Health Workers

Figure 1 shows the geographical distribution of all the places where SAM treatment is delivered. The locations of HFs are colored in orange with a buffer area of 5 km, the locations of the CHWs are in blue with a buffer area of 3 km, and the overlapped areas between providers are represented in yellow.

Table 2 provides figures on the proportion of the overlapping regions. In the Kita and Kayes districts, the overlap is approximately 50% and the duplication of services is higher in the areas closer to the district’s centers, not only between CHWs and HFs but also between HFs. The district with the lowest overlap is Bafoulabé (31%).

**Table 2:** Geospatial analysis of influential variables that can affect severe acute malnutrition treatment coverage at the three largest districts of Kayes Region in Mali

	^a^Kita District % (95% CI) High supervision	^b^Kayes District % (95% CI) Light supervision	^c^Bafoulabé District % (95% CI) Not supportive supervision	Comparison^*^ (*p* value)
Overlap between areas covered by HFs and CHWs	48.7 (39.2–58.2)	55.8 (45.3–65.9)	30.9 (19.2–44.3)	a–b: < 0.001 a–c: < 0.001 b–c: < 0.001
Children 0–59 months without geographical access to a health provider	70.4 (70.1–70.6)	52.6 (52.3–52.8)	80.4 (79.9–80.8)	a–b: < 0.001 a–c: < 0.001 b–c: < 0.001
Children 0–59 months not covered by active screening	52.2 (51.9–52.5)	9.8 (9.6–10.0)	68.6 (68.0–69.1)	a–b: < 0.001 a–c: < 0.001 b–c: < 0.001

The letters a, b and c refer to each of the districts to summarize which districts are being compared in the final comparison column

CHWs: community health workers; HFs: health facilities

**Fig. 2:**
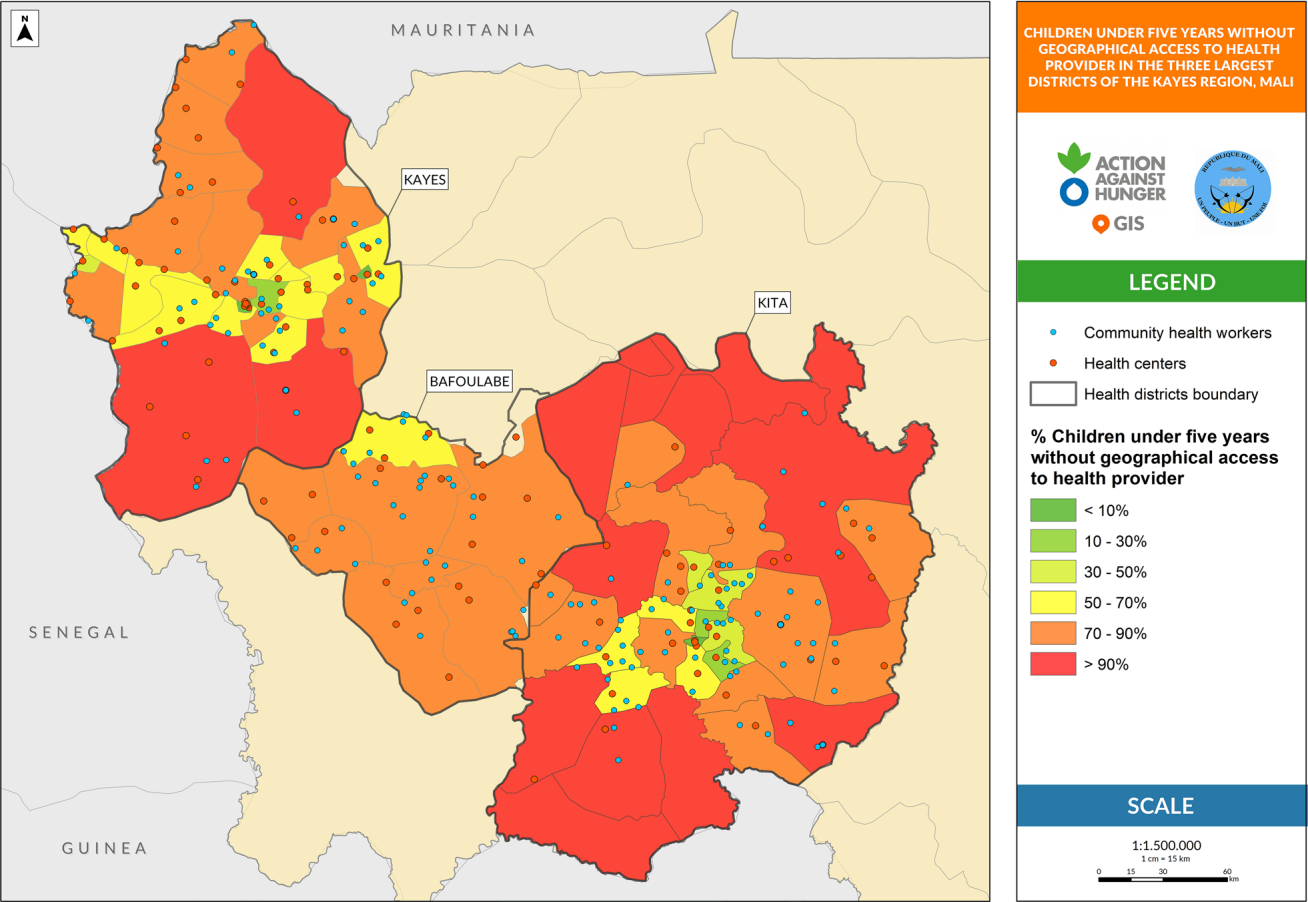
Children 0–59 months without geographical access to a health provider

The proportion of children under five years without geographical access to any health care provider is represented in Fig. 2. In this map, the red dots represent the location of the HFs, and the blue dots represent the location of CHWs. The shading of the health care areas represents the proportion of children living outside the coverage buffers of these treatment points. The red zones in the northern and southern Kita and Kayes districts show a high percentage of children without health care access.

The proportion of children without access to healthcare provider in Kita and Bafoulabé is around three-quarters of the total children population (70% and 80%, respectively), while in Kayes, this figure is approximately half of all children (52%) (Table 2).

**Fig. 3:**
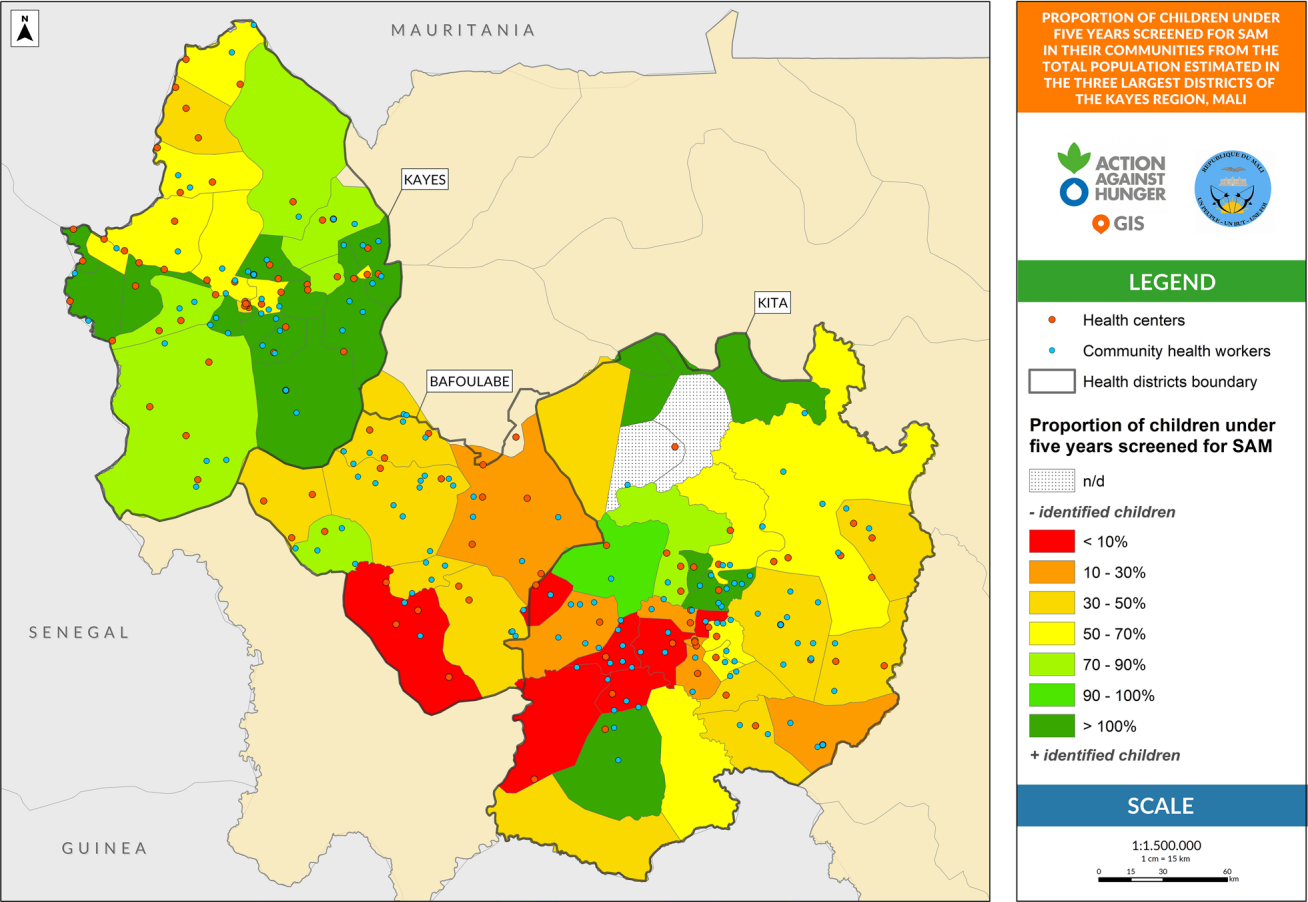
Children 0–59 months not covered by active screening for severe acute malnutrition

Figure 3 represents the proportion of children under five years screened in their communities by community health volunteers in active screening campaigns. Health areas in red and orange show the highest proportion of children not covered by screening. Most of them are located in Bafoulabé and Kita’s western regions.

The mean proportion of children not screened for SAM is shown in Table 2. Kayes District’s screening campaigns were very effective, with less than 10% of children remaining unassessed, while in Kita and Bafoulabé, it reaches more than half of the child population.

Interactive maps corresponding to Figs. 1, 2, 3 are available online for further visual analysis: https://acf-spain.maps.arcgis.com/apps/opsdashboard/index.html#/b2b854dfc1ae4ecbbf523500062cbc4d.

## Discussion

The present study shows that CHWs increased the coverage of SAM treatment in two of the three districts where decentralized treatment out of HFs was scaled-up. According to international Sphere Standards for humanitarian interventions, the proportion of SAM cases with access to treatment services in a CMAM program should reach values above 50% in rural settings [8]. This was the case in the district of Kayes and Bafoulabé. These results come to support the previous evidence available. Bliss et al. in a systematic review concluded that scaling up the use of MUAC measurement by caregivers and the use of CHWs to detect and treat SAM in community settings is a promising step toward improving the coverage of SAM treatment [18]. In another review of experiences with CHWs in urban settings between 2000 and 2018, Wahl et al. stated a need to deliver community-based interventions involving CHWs to achieve Universal Health Coverage [26]. A recent non-randomized controlled trial in Tanzania has shown the potential of CHWs in increasing coverage of SAM treatment in places where HFs are located at a long distance from households (registering 80.9% of SAM children covered with the CHWs compared to 41.7% in the control area) [27].

The different levels of supportive supervision received by the CHWs were expected to impact the quality of care and treatment outcomes. This has been confirmed in a previous publication [20]. However, the level of supervision received by the CHWs was not expected to have an impact on treatment coverage. As previously explained, the coverage surveys carried out in this region of Mali, indicate that it depends on factors related to the geographical and economic access of the affected families and the lack of knowledge about malnutrition and its consequences [7]. Since all available CHWs have been incorporated as new treatment providers in the three districts, a similar increase in coverage was expected. However, this has not been true in Kita, where treatment coverage has not changed despite adding new treatment sites with the CHWs. This result highlighted the need to analyze the optimal conditions for this intervention to achieve a relevant impact on coverage figures.

The Bafoulabé district started with the worst SAM treatment coverage figure of the three districts and increased by 40% by the end of the study. The other variables analyzed have shown that this district has the lowest overlap among its health structures. However, it has a high proportion of children without geographical access to a health provider and a high proportion of children non-covered by active screening. The Kayes district also recorded a considerable increase in coverage of 28%. It was the district where community-based screening campaigns had a higher proportion of children covered and the highest proportion of the population had access to essential health services. Unlike the other districts where at least one of the three analyzed indicators was low, Kita has high figures for all the geospatial variables analyzed. This district registered a high proportion of overlap between HFs or CHWs, a high proportion of children without geographical access to health care, and a high proportion of children non screened for SAM in their communities.

The difference concerning access to health care has previously been evidenced in Mali. Saint-Firmin et al. identified the Kayes region as the rural area with the higher percentage of people unserved by CHWs and evidenced differences inside districts. They conclude those geospatial analytics facilitate reasoning for decision-makers [28]. With a similar approach, Huerta-Munoz et al. analyzed the geographical perspective of primary health care in a rural area in Rwanda. Their study shows great variability in the geographical location and service delivery coverage and evidence that geospatial analysis programs help guide governments and policy decision-makers where resources should be targeted. They conclude the need to expand the number of CHWs and assess their best location to reduce inequity in access to health care [29]. In Mozambique, Dos Anjos et al. demonstrate that geographic access to the health care network plays an essential role in health equity. Their geospatial analysis proves that 66.7% of the population lives in unserved areas and the distance between patient and health care provider is an essential element of access to health care. They also found that creating new health provider places and reallocating existing to maximize accessibility can increase health care coverage in the country [30]. Oliphant et al. in Niger showed that the efficiency of geographical targeting of CHWs and community health posts is essential to improve access to Primary Health Care [31]. All these results are consistent with the present study, where the District of Kita, with a relatively high number of healthcare providers, registered the lower coverage of SAM treatment. These results highlight the importance of where CHWs are located, even more than the number of available health care providers.

The present study analyzes the effect of community screening in the coverage of SAM treatment. According to geospatial analysis, Kayes is the district recording the lowest proportion of children not being screened for SAM in their communities and achieving an increase of 28% in treatment coverage. The barriers and booster qualitative assessment during the coverage survey have also shown that the screening barrier was overcome after one year of the intervention. This relationship between coverage and screening has been described previously in Mali. Nyirandutiye et al. analyzed how the low coverage of screening at the community level (22% of children under five) led the Ministry of Health to include screening campaigns with MUAC tapes during National Nutrition Days to increase referrals and coverage of acute malnutrition treatment [32].

Additionally, a coverage survey carried out in Bamako listed the low coverage of screening for acute malnutrition as an important barrier to adequate CMAM treatment coverage [33]. In another study carried out in Indonesia, where low identification of cases in the community was identified as a barrier to treatment access, researchers set up an early identification strategy by community mobilization that helped increase screening from 17 to 66% in less than three years to increase SAM treatment coverage [34]. However, this argument has not been met in the Bafoulabé district, where despite having the highest number of children under five not screened in their communities (69%), it records the highest increase in coverage of SAM treatment by the end of the study (41%). Further analyses are needed to understand how different interventions implemented simultaneously can have a multiplier effect on coverage.

These results have highlighted the need to coordinate nutritional curative and preventive actions. If the decentralized model of SAM treatment with CHWs aims to increase coverage effectively, an efficient system for the early identification and referral of children suffering acute malnutrition is recommended. The WHO recommendations for CMAM interventions rely on community volunteers to conduct first-line screenings in the community and refer children identified as acutely malnourished for treatment [35]. However, alternative models involving community volunteers can be explored to achieve the goal of leaving no child unidentified. An example is the Family-MUAC approach, another of the simplified approaches identified by UNICEF to improve the effectiveness, quality, coverage, and cost-effectiveness of services to treat child wasting [36]. It consisted of the use of MUAC tapes by caregivers in their households. Several studies have shown that mothers can screen their children frequently with the proper training, allowing early diagnosis and referral for treatment. This novel approach is another significant step in scaling up acute malnutrition treatment [37, 38].

This study faced several limitations. The analyses of the influential variables that can impact coverage are based on statistical projections of the latest available demographic data, which may not accurately reflect the current situation in the area. Accordingly, the proportion of children 0–59 months without health care access is a theoretical estimate that allows us to be closer to the reality of the regions. Still, it will never be fully accurate, as the settlements are irregular and depend on many aspects that are not symmetrical. Additionally, the supply of RUTF was ensured by Action Against Hunger, so no stock-outs were registered during the study period. This may not reflect the real situation of the programs developed by the health authorities without external support. Finally, this study evidence the need for complementary analyses to evaluate the critical distance that families can walk for health care, road and path networks, the geographical barriers to access during the different seasons, as well as to measure the effect together of preventive and curative actions in the coverage of malnutrition treatment in children under five.

## Conclusions

To the best of our knowledge, this is the first study in West Africa to assess the potential of scaling up a decentralized model of SAM treatment with CHWs to increase treatment coverage. The results suggest that involving CHWs as SAM treatment providers outside the HFs can considerably increase treatment coverage. However, policymakers may consider other critical aspects before establishing decentralized treatment at a country level as the geographical location of the CHWs to avoid overlap among health providers and ensure the coverage of all unserved areas according to their population densities. At the same time, other simplified approaches, such as the identification and screening through the Family-MUAC, can be implemented in addition to CHW-led treatment to increase the impact that this model of intervention can have in the fight against acute malnutrition.

## Data Availability

The datasets used and/or analyzed during the current study are available from the corresponding author on reasonable request.
